# Challenges in Managing a Congenital Strangulated Bochdalek Hernia in an Older Female Patient in a Low-Income Country: A Case Report

**DOI:** 10.3389/jaws.2025.15289

**Published:** 2025-12-08

**Authors:** Jacques Noel Tendeng, Guillaume Tcheutchoua Soh, Diago Anta Dia, Franck Armel Tene Nde, Abdourahmane Ndong, Philippe Manyacka Ma Nyemb, Ibrahima Konate

**Affiliations:** Department of Surgery, Gaston Berger University, Saint-Louis, Senegal

**Keywords:** Bochdalek hernia, COVID-19, strangulation, low-income country, adult

## Abstract

**Introduction:**

Bochdalek hernias are the most common type of congenital diaphragmatic hernia. They can remain asymptomatic throughout life. Occasionally, however, they are discovered when complications arise, such as respiratory distress due to strangulation or volvulus of herniated organs. A late diagnosis is common due to their rarity in older adults. This condition needs early diagnosis, treatment, and strong supportive care to save the patient’s life. In this study, we discuss the specific challenges encountered when managing a congenital strangulated Bochdalek hernia in an older female patient in our tertiary healthcare centre.

**Case Presentation:**

We present the case of an 88-year-old Black woman who was admitted to our emergency department in October 2021. She had a history of epigastric pain and hypertension. She was admitted for dyspnoea, constipation and abdominal pain lasting 24 h. At this time, it was a unique diagnostic we suspect due to the epidmiology context. Initial investigations included a rapid COVID-19 test, a PCR test for COVID-19, and a chest X-ray. The former two were negative, but the latter showed a gastric pocket in the left chest. We suspected a diaphragmatic hernia. The following day, the patient underwent thoracoabdominal computed tomography, which revealed a left diaphragmatic hernia with signs of intestinal necrosis, suggesting strangulation. An electrocardiogram showed sinus tachycardia, and an ultrasound revealed a heart with normal left ventricular function. The patient underwent emergency laparotomy, which revealed intestinal necrosis. The spleen was found to be lacerated, and the stomach had ascended into the left thoracic cavity. The gangrenous ileum was resected with loop stoma formation, the spleen was removed, and the hernia orifice was repaired by interrupted stitches. Unfortunately, the patient died on the fourth post-operative day due to Multiple Organ Dysfunction Syndrome.

**Conclusion:**

Strangulated Bochdalek hernia is a rare emergency condition with non-specific signs. It requires rapid and comprehensive investigation to avoid delays in diagnosis. Surgery is the definitive treatment. In older adults, successful management depends on the presence of morbidities that may not be fully addressed before surgery, intensive and rapid perioperative resuscitation, and emergency surgery. The triage process during the pandemic may also have contributed to a worse prognosis, especially in low-income countries.

## Introduction

Congenital diaphragmatic hernias are conditions that are diagnosed during the perinatal period. Bochdalek hernia is the most common type [1]. The majority of Bochdalek hernias are asymptomatic in adults. Some patients with Bochdalek hernias may be diagnosed incidentally with vague or minimal symptoms [2]. They can rarely be revealed by complications, such as respiratory distress, strangulation, gastric volvulus, sudden death and/or intestinal occlusion [3–5]. In the event of strangulation, the symptoms are non-specific. The diagnosis is suspected based on X-rays and computerised tomography scans. Surgery is the definitive treatment for this condition. It is a potentially highly lethal condition, with mortality rates depending on the centre where the patient is treated. It is an emergency condition requiring early diagnosis, treatment, and strong supportive care to save the patient’s life [5]. In this study, we present a case of congenital strangulated Bochdalek hernia in an older female patient who was treated at our tertiary healthcare centre, and we discuss the challenges encountered. This case was reported in accordance with the CARE 2013 guidelines [6].

## Case Presentation

**FIGURE 1:**
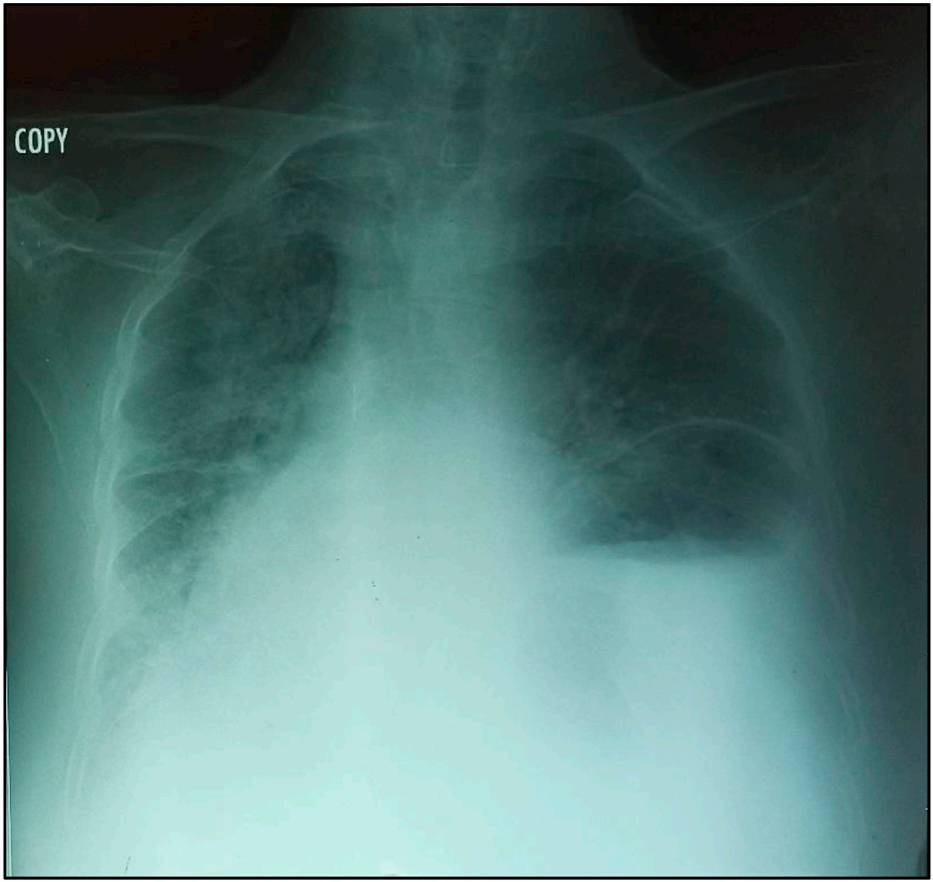
Chest radiography showing enlargement of the mediastinum and the gastric pocket in the left chest.

**FIGURE 2:**
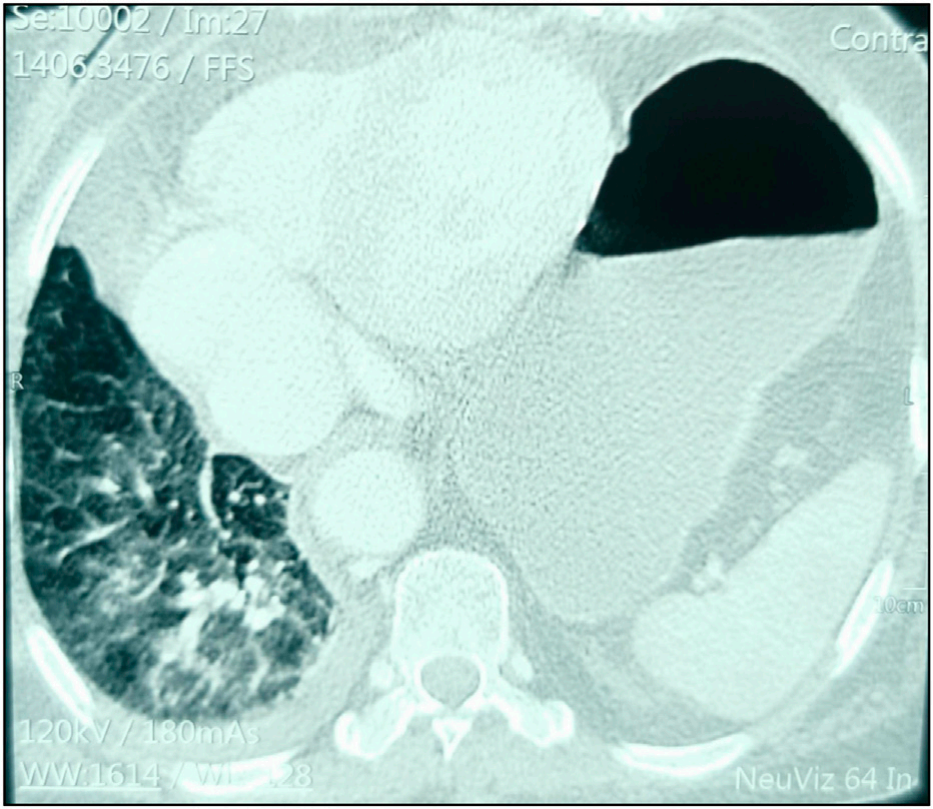
Chest computed tomography showing the spleen and stomach in the left thoracic cavity and condensation of the right lung associated with the mid-pleura.

**FIGURE 3:**
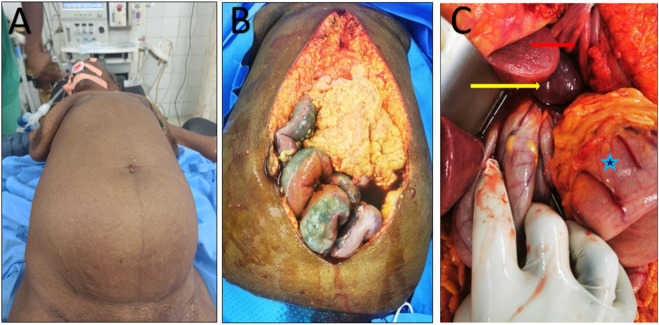
**(A)** Distended abdomen; **(B)** Necrotic ileum; **(C)** Stomach (blue star) and spleen (yellow arrow) in the left diaphragmatic defect (red arrow).

In October 2021, an 88-year-old Black woman presented with a history of hypertension and epigastric pain for 30 years. She had no history of thoracic or abdominal trauma. She was admitted with respiratory distress, constipation, and abdominal pain lasting 24 h. On admission, her Glasgow coma score was 15/15, her oxygen saturation was 89% in ambient air, she had a fever of 38.5 °C, her blood pressure was 110/90 mmHg, her heart rate was 111 beats per minute, her respiratory rate was 32 cycles per minute, and she had a distended, soft abdomen without tenderness (Figure 3A). Paraclinical examinations revealed leukocytosis of 12,000 cells per IU, with neutrophil predominance associated with lymphopenia at 700 cells/IU. The C-Reactive Protein level was 192 mg/L. At this time, it was a unique diagnostic we suspect due to the epidmiology context. Initial investigations included a rapid COVID-19 test, a PCR test for COVID-19, and a chest X-ray. The former two were negative, but the latter showed a gastric pocket in the left chest (Figure 1). We suspected a diaphragmatic hernia. The following day, the patient underwent thoracoabdominal computed tomography, which revealed a left posterolateral diaphragmatic hernia with signs of intestinal necrosis, suggesting strangulation; the PCR test for confirmation of a SARS-CoV-2 infection was negative. Other anomalies seen during the CT scans included the spleen and stomach in the left chest, condensation in the right lung, and right pleural effusion (Figure 2). An electrocardiogram showed sinus tachycardia, and a heart ultrasound showed dextrocardia with a normal left ventricular ejection fraction. Initial resuscitation consisted of hydration with normal saline, insertion of a nasogastric tube, and a Foley catheter. The patient then underwent a mid-laparotomy performed by two senior consultants in general surgery, which revealed a 7-cm left Bochdalek hernia orifice containing the stomach and the spleen which had been accidentally lacerated, and 1 m of necrotic ileum (Figures 3B,C). The herniated contents were returned to the abdominal cavity, the hernia orifice was repaired with interrupted non-absorbable stitches, and a splenectomy and resection of the necrotic ileum with loop stoma formation were performed. The chest and abdomen were drained. The patient was admitted to the intensive care unit (ICU) on mechanical ventilation. She received a course of ceftriaxone (4 g/day) and metronidazole (500 mg/8 h) in addition to other supportive care. She was intubated until the third post-operative day, and on that day, she experienced progressive multiorgan dysfunction, including haematological, renal, and central nervous system dysfunction. She died on the fourth day after the surgery.

## Discussion

Diaphragmatic hernias are a well-known congenital defect in children, with an incidence of 1 in every 4,000-5,000 births [7]. The most frequent type of diaphragmatic hernia is the Bochdalek hernia. Its estimated incidence in the global population is 0.17%–6% [1, 4, 8, 9]. Historically, symptomatic and strangulated diaphragmatic hernias have been rarely reported in the literature [10]. As in our case, these are more frequent on the left diaphragm due to the early closure of the pleuroperitoneal canal on the right side [10, 11].

Presenting symptoms are usually nonspecific and may include respiratory or digestive symptoms, such as abdominal pain, abdominal distension, vomiting, and constipation. These are more common in older children and older adults, as observed in our patient [5]. These signs suggests obstruction, and there are no specific clinical signs of strangulation. In our case, differential diagnoses were a) a possible link with the epidemiological context of 2021 and a potential diagnosis of a SARS-CoV-2 infection; b) diaphragmatic eventration; c) pulmonary abscess; and d) tension pneumothorax [1, 4, 12]. However, it was difficult to establish an association between Bochdalek hernia and a SARS-CoV-2 infection in our case, given that Bochdalek hernias are rare in adults [9]. All patients presenting to the emergency unit with chest symptoms during the time of the pandemic were considered to be infected with COVID-19 until proven otherwise [4, 13, 14]. This measure can delay the diagnosis of other diseases, especially in sub-Saharan Africa, where lower-level resources are available, such as CT scans, X-rays, and blood tests. Also they experience redistribution of inputs with a longer adaptation period. This impact was observed during the management of our patient. Usually, the diagnosis of diaphragmatic eventration can be made with a chest X-ray and confirmed with a chest CT scan [9]. CT scans have the advantage of enabling the hernia to be characterised more accurately in terms of its type, content, and associated anomalies, in addition to showing signs of complications. In our case, these were signs of intestinal necrosis, pleural effusion, and pulmonary condensation [10]. Necrosis of the large bowel was also observed on CT in a case reported by Das et al [11]. Evidence of necrosis of the viscera on a CT scan is an obvious sign of strangulation.

We performed a midline laparotomy due to the presence of signs of intestinal necrosis on the CT scan and the general state of the patient. An abdominal approach is preferred in cases of obstruction or strangulation [9, 10]. After reinsertion of the viscera, a splenectomy and diaphragmatic hernia repair were performed. The safest procedure was resection of the necrotic ileum, followed by ileostomy formation, and drainage of both the thoracic and abdominal cavities. This is recommended by the World Society of Emergency Surgery in the event of a diaphragmatic hernia with intraoperative instability [15]. In our case, the presence of strangulation, advanced age, hypertension, intestinal necrosis, a lacerated spleen, and signs of pneumonia and pleural effusion on the right chest resulted in a poor prognosis. All of these factors highlight the need for strong supportive perioperative care and surgery. The procedure has a better outcome in children compared to neonates [16]. The outcomes are inconsistent in the literature [15]. Machado et al. have recorded a 2.7% mortality rate for laparoscopic surgery in their study, which included Bochdalek hernias in adult patients [17]. Mortality varies from 14.3%–20% [15]. This condition represent a potentially high-mortality emergency. DeAlwis et al have reported a case of sudden death due to a hernia, and Manson et al have reported cardiac arrest due to a congenital Bochdalek hernia, highlighting the challenges involved in the management of acute complications of this condition [5, 18].

In conclusion, we believe that an extensive study, such as a systematic review, is needed to better identify the factors associated with successful treatment, estimate the worldwide incidence and management of this disease, and establish guidelines for its treatment despite it being rare.

## Conclusion

Strangulated Bochdalek hernia is a rare emergency condition in adults with nonspecific symptoms. It requires a rapid and comprehensive initial assessment to avoid delays in diagnosis. Surgical intervention is the definitive treatment. In older adults, successful management depends on the presence of morbidities that may not be fully addressed before surgery, intensive and rapid perioperative resuscitation, and emergency surgery. The triage process during the pandemic may have contributed to a worse prognosis, especially in low-income countries.

## Data Availability

The raw data supporting the conclusions of this article will be made available by the authors, without undue reservation.
